# Night Sleep, Parental Bedtime Practices and Language Development in Low-Risk Preterm and Full-Term Late Talkers: A Longitudinal Study in the Third Year of Life

**DOI:** 10.3390/children11111393

**Published:** 2024-11-16

**Authors:** Mariagrazia Zuccarini, Martina Riva, Arianna Aceti, Luigi Corvaglia, Anat Scher, Annalisa Guarini, Alessandra Sansavini

**Affiliations:** 1 Department of Education Studies “Giovanni Maria Bertin”, University of Bologna, Via Filippo Re 6, 40126 Bologna, Italy; mariagrazia.zuccarini@unibo.it; 2 Department of Psychology “Renzo Canestrari”, University of Bologna, Viale Berti Pichat 5, 40127 Bologna, Italy; martina.riva4@studio.unibo.it (M.R.); annalisa.guarini@unibo.it (A.G.); 3 Department of Psychology, University of Milano-Bicocca, Piazza dell’Ateneo Nuovo 1, 20126 Milano, Italy; 4 Neonatal Intensive Care Unit, IRCCS Azienda Ospedaliero-Universitaria di Bologna, Via Massarenti 9, 40138 Bologna, Italy; arianna.aceti2@unibo.it (A.A.); luigi.corvaglia@unibo.it (L.C.); 5 Department of Medical and Surgical Sciences, University of Bologna, via Massarenti, 9, 40138 Bologna, Italy; 6 Department of Counseling and Human Development, University of Haifa, Abba Khoushy Ave 199, Haifa 3498838, Israel; anats@edu.haifa.ac.il

**Keywords:** night sleep, parental bedtime practices, expressive language, late talkers, preterm birth

## Abstract

*Background*: Studies on night sleep and parental bedtime practices and their associations with language development in populations at risk of language delay and neonatal conditions, such as late talkers and preterm children, are scarce. *Objectives*: Our objective was to longitudinally examine the development of night sleep (total night sleep difficulties, settling, night waking, and co-sleeping), parental bedtime practices (total parental bedtime practices, active physical comforting, encouraging autonomy, and leaving to cry), and expressive language (word and sentence production), and their associations in low-risk preterm and full-term late talkers from 31 to 37 months of age. *Methods*: Parents of 38 late talkers, 19 low-risk preterm and 19 full-term children, completed the Italian versions of the Infant Sleep Questionnaire, the Parental Interactive Bedtime Behavior Scale, and the MacArthur-Bates Communicative Development Inventory Words and Sentences Long Form. *Results*: Late talkers’ night sleep difficulties, such as settling to sleep and night waking, decreased over time, with low-risk preterm late talkers experiencing more night waking and co-sleeping than full-term peers. Parents reported that instances of active physical comforting and leaving to cry also decreased, with parents of low-risk preterm late talkers reporting higher active physical comforting scores than parents of full-term peers. Improvements in parental practices of encouraging autonomy were significantly associated with increased sentence production from 31 to 37 months. *Conclusions*: Findings highlight the importance of monitoring night sleep in preterm and full-term late talkers. They also suggest that populations vulnerable to sleep and language delays may particularly benefit from targeted interventions promoting autonomy in their bedtime routines, which, in turn, could support their language development trajectories.

## 1. Introduction

### 1.1. Night Sleep in Typical and Atypical Development

Sleep patterns undergo significant changes throughout a lifespan, with the early years of life marked by substantial advances in sleep organization, both during the day and at night [1,2,3,4,5]. During the first three years of life, a notable increase in night sleep duration occurs, characterized by a non-linear trajectory, with decreasing night waking, increased sleep efficiency, and greater stability in the time to fall asleep [1,4,5]. However, sleep consolidation with stable sleep patterns is only achieved during the school years [6,7].

Regarding atypical developmental trajectories, studies on children with neurodevelopmental disorders (i.e., Down syndrome, Fragile X syndrome, Williams syndrome) showed more night sleep problems compared to typically developing children [8,9]. Notably, Halstead et al. [9] found that children with neurodevelopmental disorders had higher rates of sleep disorders compared to other clinical samples of children. In addition, sleep disturbances in children with neurodevelopmental disorders may contribute to the exacerbation of their specific phenotype, highlighting the importance of investigating and addressing night sleep issues in atypically developing populations [8,9].

As regards at-risk populations, particularly those at risk of language delay, such as late talkers, studies on night sleep are scarce, with preliminary and inconsistent results. Late talkers are 2- to 3-year-old children with an expressive vocabulary size ≤ 10th percentile, assessed through parental questionnaires (e.g., the MacArthur-Bates Communicative Development Inventories, MB-CDIs, [10]), and without neurological disorders, intellectual disabilities, or sensory impairments [11]. Zubrick and colleagues [12] did not find any significant difference in sleep patterns between late talkers and typically developing children. In contrast, Sansavini and colleagues [13] found difficulties in settling at night, frequent night waking and co-sleeping in late talkers. Noteworthy, those two studies explored sleep difficulties through two different parental questionnaires. Zubrick et al. [12] employed the Child Behavior Checklist (CBCL; [14]), listing 99 specific behavior problems, including those related to sleep, that investigate the presence of sleep disorders, such as recurring nightmares or crying and screaming during sleep. Sansavini et al. [13] employed the Infant Sleep Questionnaire (ISQ; [15]), assessing sleep-related behaviors (e.g., settling, night waking, co-sleeping). In addition, no studies have taken a longitudinal approach to investigating changes in night sleep in this population.

Preterm birth, i.e., a birth occurring before 37 weeks of gestation, has been identified as one of the factors increasing the risk of language delay [16]. There is some evidence that preterm birth is associated with poor sleep quality [17,18,19]; however, findings are only partially consistent. Some studies have not found significant differences in sleep quality and duration between preterm and term-born children [20,21], whereas other studies have identified preterm birth as a predictor of sleep characteristics [13,22]. These discrepancies might partly depend on the different tools used to measure sleep: actigraphy [17], a face-to-face parent interview on child sleep behaviors over the last three months [20], or parental questionnaires [13,18,19,21,22]. A further reason might be related to the different characteristics of the preterm samples included in the studies, as some of them [13,17,18,19,22] included preterm infants with a mean gestational age ≤33 weeks and/or gestation-related risk (i.e., low birth weight, small for gestational age), whereas others [20,21] included preterm children with a mean gestational age between 34 and 36 weeks and no specific gestation-related risk. These conflicting findings highlight the need for further research to elucidate the impact of preterm birth on sleep development. In addition, studies focusing on night sleep in late talkers that consider neonatal condition (i.e., preterm vs. full-term birth) are lacking, except for one study showing that preterm late talkers experience more sleep difficulties, particularly night waking, than full-term late talkers [13].

### 1.2. Parental Bedtime Practices in Typical and Atypical Development

Parental bedtime practices [4,23] are among the important factors that shape sleep in early years [24]. These practices may play a significant role in facilitating children’s sleep onset and managing night waking [23,25]. Sleep-related parenting behaviors vary depending on family factors (e.g., parental educational level, socioeconomic status, stress, and sleep beliefs), contextual factors (e.g., culture), and infant factors (e.g., temperament, developmental characteristics, sleep characteristics, and age) [4,13,25,26,27]. Morrell and Cortina-Borja [23] identified several types of parental bedtime practices that are dynamically modified by parents to accommodate the infant’s developmental needs. Active physical comforting and movement-based parental bedtime practices decrease during the first three years, whereas other strategies, such as encouraging autonomy, become more prevalent, promoting night sleep consolidation [4,23].

Concerning the use of these practices in populations at risk of language delay, Sansavini et al. [13] found that parents of 30-month-old late talkers, like parents of typically developing children at the same age range [23], frequently used practices encouraging autonomy. In Sansavini’s study, preterm birth did not appear to affect the parental bedtime practices of late talkers [13].

### 1.3. Relationships Between Night Sleep, Parental Bedtime Practices, and Language Development

Sleep patterns and their developmental trajectories play a crucial role in shaping children’s overall well-being and development in several domains, including cognition, memory, learning, emotions, and academic achievement [2,7,27,28]. Concerning language development, several studies have reported a link between early childhood sleep consolidation and quality and subsequent linguistic outcomes [3,29,30,31,32,33,34]. For example, two recent studies on typically developing children showed that children with longer and more consistent night sleep trajectories over the first two years of age had better receptive and expressive language skills, as assessed with the Bayley-III scales [35], at 24 months [29,34]. Multivariate analyses showed that these predictive associations persisted even when controlling with possible confounding factors such as language score at 1 year of age, or maternal depressive symptoms at 24 months of age [29,34]. Positive associations between night sleep regulation and language development, assessed with standardized tools, were found until 36 months of age [29] and up to preschool and school-age [29,30]. This evidence suggests that night sleep has a long-lasting impact on language development. In addition, sleep appears to be a relevant factor in facilitating the acquisition of new words and their generalization, as well as the grammatical patterns of language. Indeed, some studies have shown that, compared to children who stayed awake, children who had napped better remembered linguistic stimuli they were exposed to 1.5, 2, or 4 h later [36,37,38].

A link between night sleep and language development was also found in D’Souza et al.’s study on children with neurodevelopmental disorders [8]. Those authors found that, after accounting for age, night sleep time explained additional variance in the receptive vocabulary size of children with Down and Williams Syndrome aged 9 to 55 months. Specifically, for every two minutes of night sleep time, these children’s language comprehension increased by one word [8]. Those findings suggest that sleep may influence language development in atypically developing children; however, the scarcity of evidence on this matter calls for further research. Interestingly, a study by Dionne and colleagues [39] has shown that children with language disorder at 60 months of age had less mature sleep consolidation in the first two years of age. This finding underscores the importance of considering sleep patterns in the study of language development in populations at risk of language delay, such as late talkers, and is in line with the neuroconstructivistic theoretical framework of language development, which posits that language is shaped by diverse, interconnected, and interdependent processes and abilities [40].

Fiese et al. [41] also found that language-based bedtime routines during the first two years of life were associated with better sleep outcomes. More language-based routines at 12 months predicted fewer sleep problems at 18 months and were associated with longer sleep duration at 18 and 24 months [41]. These routines, which become more common as the child’s age increases, were shown to be beneficial for both sleep quality and language development [26,27,42]. Findings of a positive predictive association between language-based routines at bedtime at three years of age and language scores at five years of age have been documented, with children who are regularly exposed to language-based routines at three years of age reporting higher scores in language skills at five years of age, even when controlling for linguistic scores at 36 months [26]. Along these lines, a study by Williams and Horst [42] has shown that shared book reading before falling asleep promoted word acquisition in typically developing 3-year-old children; they remembered more novel words 2.5 h later, 24 h later, and 7 days later compared to 3-year-olds who did not nap [42]. Overall, this research underscores the significance of studying the implications of parental bedtime practices for language development. In addition, very little is known about this association in populations at risk of language delay and neonatal condition. As previously mentioned, according to the neuroconstructivistic approach, integrating data from different domains besides language, such as sleep and parental behaviors related to sleep, might contribute to understanding the mechanisms that drive language development and help to implement more effective interventions for cases in which language development is atypical [40].

### 1.4. Aims

This study aimed to bridge the gaps in the literature mentioned above concerning the development of night sleep, parental bedtime practices, and language development and their relationships in infants at risk of language delay, integrating data from different domains, at different levels, and across time [40]. We tried to embrace the topic’s complexity by investigating the development of night sleep perceived by parents (i.e., total night sleep difficulties, settling, night waking, and co-sleeping), parental bedtime practices (i.e., total parental bedtime practices, active physical comforting, encouraging autonomy, and leaving to cry), and expressive language development (i.e., word and sentence production) in late talkers during the third year of life (from 31 to 37 months of age). We also looked at data that considered the role of neonatal condition (low-risk preterm vs. full-term birth). A further aim was to investigate the associations between changes in night sleep, parental bedtime practices, and expressive language from 31 to 37 months of age.

Regarding the development of late talkers’ night sleep and parental bedtime practices between 31 and 37 months of age, we expected a decrease in the night sleep difficulties of late talkers, but also the persistence of more perceived night sleep difficulties and more physically active parental bedtime practices in low-risk preterm compared to full-term late talkers. In addition, we expected an increase in the word and sentence production of late talkers during this time period, independently of neonatal condition.

Concerning the relationships among changes in night sleep, parental bedtime practices, and expressive language between 31 and 37 months, taking into account previous research on typically and atypically developing populations, we expected that a change in night sleep would be positively associated with a change in word and sentence production in late talkers. As regards parental bedtime practices, we expected that changes in encouraging autonomy would be positively associated with a change in word and sentence production in late talkers.

## 2. Methods

### 2.1. Participants

Thirty-eight late talkers participated in this study. All children were recruited at the Policlinico di Sant’Orsola Hospital of the IRCCS Azienda Ospedaliero-Universitaria di Bologna, Italy, Italy. The criteria for inclusion were as follows: (a) being monolingual or predominantly exposed to Italian (over 65% of daily language exposure) from birth; (b) having no major brain injuries, congenital anomalies, or visual, auditory, or motor impairments (for more details see [13]); and having no significant cognitive impairments (identified by a BSID-III cognitive score <70).

The sample included 19 low-risk preterm children born before 37 weeks of gestational age and 19 full-term children with a gestational age ≥37 weeks. They were longitudinally assessed for language delay at 31 months (*M* = 31.41; *SD* = 1.39) and 37 months of age (*M* = 37.58; *SD* = 1.59) at the Developmental Psychology Lab of the Department of Psychology “Renzo Canestrari”, University of Bologna, Italy. For low-risk preterm children, age was adjusted based on weeks of prematurity to account for their neurobiological maturation level [13,43].

The low-risk preterm and full-term children had similar socio-demographic characteristics, except for a higher rate of non-Italian fathers in the low-risk preterm group, whereas, as expected, they differed for some birth conditions (see Table 1). Indeed, low-risk preterm children had a lower gestational age and birth weight and a higher number of hospitalization days and prevalence of caesarean births and twin births. They also had some medical complications, such as respiratory distress syndrome and hyperbilirubinemia, but not severe ones, as they were low-risk preterm. The study complied with ethical standards for the protection of human subjects and with General Data Protection Regulation (GDPR 2016/679) and obtained formal approval from the Bologna Health Authority’s Independent Ethics Committee (protocol numbers: EM 194-2017__76/2013/U/Sper/AOUBo, EM 193-2018_76/2013/U/Sper/AOUBo, EM1229-2020_76/2013/U/Sper/AOUBo). Informed written consent was signed by parents for participation in the study, data analysis, anonymous data publication, and protection of personal data.

### 2.2. Tools

#### 2.2.1. The Italian Version of the MacArthur-Bates Communicative Development Inventories (MB-CDIs), Words and Sentences Long Form

We evaluated linguistic abilities using the Italian version of the MacArthur-Bates Communicative Development Inventories (MB-CDIs), Words and Sentences Long Form [45]. In this study, we employed the first and the third section of the questionnaire. The first section measures expressive vocabulary through a 670-word checklist. Parents marked each word their child could spontaneously produce. Each checked word scored 1 point; the total score represented the child’s overall word production.

The third section assesses expressive syntax with 37 sentence pairs. For each pair, parents selected the sentence form (either incomplete or complete with function words) that best matched their child’s speech. Each selection was scored with 1 point, and these were summed to calculate the child’s total sentence production score.

#### 2.2.2. Infant Sleep Questionnaire (ISQ)

Night sleep difficulties were assessed through the Infant Sleep Questionnaire (ISQ), a parental ten-item questionnaire assessing sleeping behaviors in infants and toddlers [15]. This is a valid and reliable tool for assessing night sleep difficulties at this age [15]. In the current study, we used the Italian version of the ISQ (see Supplementary Materials SA), which has already been used in a previous study describing night sleep in late talkers [13]. Parents were requested to describe their child’s sleep behaviors by rating them on a Likert scale. According to the aims of the current study, we considered only specific items of the questionnaire. For settling, we used ISQ 1 to assess average settling latency (range 0–6) and ISQ 2 for the frequency of settling difficulties per week (range 0–7). For night waking, ISQ 4 measured the frequency of night wakings per week (range 0–7), ISQ 5 assessed the number of wakings per night (range 0–5), and ISQ 6 evaluated sleep latency after night wakings (range 0–6). For co-sleeping, ISQ 8 captured the frequency of co-sleeping due to child distress (range 0–7). We calculated an overall score by summing all items to create an index of the child’s total night sleep difficulties (ISQ total score).

We assessed the internal validity of the questionnaire with Cronbach’s alpha (α = 0.79). We also calculated subscale scores: settling by summing ISQ 1–2 (range 0–13; α = 0.80) and night waking by summing ISQ 4–5–6 (range 0–18; α = 0.76). The co-sleeping score was based on ISQ 8 (range 0–7).

#### 2.2.3. Parental Interactive Bedtime Behavior Scale (PIBBS)

We examined parental bedtime practices using the PIBBS questionnaire, which assesses various strategies parents use to help their child fall asleep during the first three years of life [23]. We used the Italian version of the PIBBS (see Supplementary Materials SB) already employed in a previous study involving late talkers [13]. This questionnaire consists of 17 items (for more details see [23]). Parents were required to rate the behaviors they currently used to settle their child off to sleep on a five-point scale from never (score = 0) to very often (score = 4). We summed up the scores of all these items to obtain a total score of the behaviors adopted by parents for settling their child (PIBBS total score). We checked the internal validity of the questionnaire of the current study with Cronbach’s alfa (*α* = 0.73 [23]). In addition, as in previous studies [13], we grouped items into two main subscales describing different parental bedtime practices: active physical comforting (items 1, 2, 3, 4, 5, 10, 12, 15, 17), where parents believe they have to actively settle their child to sleep, and encouraging autonomy (items 6, 7, 8, 9, 11, 14, 16), where parents encourage the child to settle themself. Item 13 (“leaving to cry”) was not included in any of the subscales, and it was separately examined since it did not involve any intervention from the parent. We assessed the internal validity of the aforementioned subscales, finding Cronbach’s alpha values of α = 0.73 for active physical comforting and α = 0.68 for encouraging autonomy.

#### 2.2.4. Bayley Scales of Infant and Toddler Development (BSID-III)

Children’s cognitive and language skills were evaluated using the Italian version of the Bayley Scales of Infant and Toddler Development—Third Edition (BSID-III [35,46]). Cognitive and language composite scores (*M* = 100; *SD* = 15) for each child were calculated according to the Italian normative values [46].

### 2.3. Procedure

Children identified as late talkers in a screening project (see [43] for more details) were invited, along with their parents, to the Development Psychology Lab, at the Department of Psychology “Renzo Canestrari”, University of Bologna, for a direct assessment of their cognitive abilities using the BSID-III [46]. During this visit, parents filled in MB-CDI Words and Sentences Long Form, the ISQ, and the PIBBS questionnaires. About 6 months later (around 37 months of age), parents were asked to fill out the same questionnaires to assess children’s change in night sleep, parental bedtime practices, and language skills. The change was calculated by computing the difference, for each tool and scale, between the scores obtained at 37 months and those obtained at 31 months.

### 2.4. Statistical Analyses

All analyses were performed using IBM SPSS Statistics for Windows, Version 28.0 (IBM Corp., Armonk, NY, USA). The statistical tests were bilateral with a 0.05 alpha level of significance. The normality of distribution was checked through the Kolmogorov–Smirnov test, skewness, and kurtosis, and most variables were normally distributed.

For Aim 1, repeated-measure analyses of variances were conducted to evaluate the effect of age (31 vs. 37 months) and group (low-risk preterm vs. full-term birth) on night sleep (ISQ total score, settling, night waking, and co-sleeping, see Table 2), parental bedtime practices (PIBBS total score, active physical comforting, encouraging autonomy, and leaving to cry, see Table 3), and expressive language skills (word production and sentence production, see Table 4). Bonferroni’s method was used for post hoc comparisons to account for multiple comparisons.

Concerning Aim 2, firstly, we computed the change (delta) in total night sleep difficulties perceived (ISQ total score, settling, night waking, and co-sleeping; see Table 2), parental bedtime practices (PIBBS total score, active physical comforting, encouraging autonomy, leaving to cry; see Table 3), and expressive language skills (word production and sentence production, see Table 4) by computing the difference between the scores reported at 31 and 37 months. Subsequently, we conducted Pearson’s correlational analyses to explore the associations between the deltas of night sleep and word and sentence production, and between the deltas of parental bedtime practices and word and sentence production.

## 3. Results

### 3.1. Development of Night Sleep, Parental Bedtime Practices, and Expressive Language Skills from 31 to 37 Months

Descriptive data on night sleep, parental bedtime practices, and expressive language skills at 31 and 37 months, and their deltas between 31 and 37 months, are reported in Table 2, Table 3, and Table 4, respectively.

**Figure 1:**
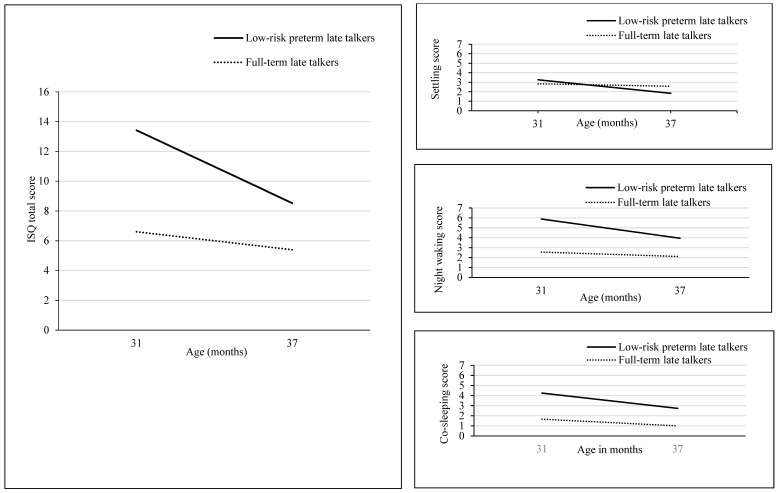
Longitudinal trajectories of mean ISQ scores (total score, settling, night waking, and co-sleeping) in low-risk preterm and full-term late talkers at 31 and 37 months.

Regarding the ISQ total score, repeated measure ANOVAs yielded significant effects of age [*F*(1, 35) = 8.98, *p* = 0.005, *η_p_*^2^ = 0.204] and group [*F*(1, 35) = 8.18, *p* = 0.007, *η_p_*^2^ = 0.189], revealing that the ISQ total score significantly decreased in late talkers between 31 and 37 months, with low-risk preterm late talkers having significantly higher scores than full-term late talkers (see Table 2 and Figure 1).

Analyzing the ISQ subscales, repeated measure ANOVAs showed an age effect for settling [*F*(1, 36) = 6.37, *p* = 0.016, *η_p_*^2^= 0.150] and night waking [*F*(1, 35) = 4.21, *p* = 0.048, *η_p_*^2^ = 0.107], showing that the amount of time needed for settling the child and the number of night wakings significantly decreased from 31 to 37 months of age in late talkers. In addition, a group effect on night waking [*F*(1, 35) = 6.44, *p* = 0.016, *η_p_*^2^ = 0.155] and co-sleeping [*F*(1, 35) = 8.56, *p* = 0.006, *η_p_*^2^ = 0.196] was found, with significant higher scores in low-risk preterm than full-term late talkers (see Table 2 and Figure 1).

As regards the PIBBS total score, repeated measure ANOVAs yielded no significant effects (see Table 3). As regards PIBBS subscales, repeated measure ANOVAs showed an age effect on both active physical comforting [*F*(1, 36) = 4.82, *p* = 0.035, *η_p_*^2^ = 0.118] and leaving to cry [*F*(1, 36) = 6.96, *p* = 0.012, *η_p_*^2^ = 0.162] scores, showing that these practices significantly decreased in late talkers from 31 to 37 months (see Table 3). In addition, a group effect on active physical comforting [*F*(1, 36) = 4.28, *p* = 0.046, *η_p_*^2^ = 0.106] was found, with significantly higher scores in low-risk preterm than full-term late talkers (see Table 3).

Concerning expressive language skills, repeated measure ANOVAs revealed a significant age effect on word production [*F*(1, 36) = 41.49, *p* < 0.001, *η_p_*^2^ = 0.535] and sentence production [*F*(1, 36) = 38.96, *p* < 0.001, partial *η_p_*^2^ = 0.520], indicating a significant increase from 31 to 37 months in late talkers (see Table 4).

### 3.2. Associations Among Changes in Night Sleep, Parental Bedtime Practices, and Expressive Language Between 31 and 37 Months

Concerning the second aim of the study, correlation analyses revealed no significant relationships between the deltas of night sleep and word and sentence production (see Table 5). Conversely, significant positive associations were found between the deltas of parental bedtime practices (total score and encouraging autonomy score) and sentence production (see Table 5).

## 4. Discussion

The present study investigated night sleep and parental bedtime practice trajectories and their association with language development during the third year of life, focusing on the at-risk population of late talkers and considering the role of neonatal condition (low-risk preterm vs. full-term birth).

Our findings provided valuable insights into how night sleep and parental bedtime practices develop in this population. The results showed that late talkers’ night sleep difficulties, active physical comforting, and leaving to cry bedtime practices decreased during the third year of life in both low-risk preterm and full-term late talkers. However, night sleep difficulties and active physical comforting bedtime practices were persistently higher in low-risk preterm than full-term late talkers.

In addition, the association found between changes in parental bedtime practices, specifically in those encouraging autonomy and in sentence production in late talkers, shed light on the complex interplay between these aspects and suggested potential avenues for intervention supporting consolidated and well-regulated night sleep, age-appropriate parental bedtime practices, and language development in this at-risk population.

### 4.1. Night Sleep, Parental Bedtime Practices, and Expressive Language Development in Low-Risk and Full-Term Late Talkers

Our results revealed that night sleep difficulties, specifically the amount of time needed for settling the child and the number of night wakings, decreased in late talkers from 31 to 37 months of age. These findings demonstrate that night sleep efficiency increases and night waking decreases as late-talking children grow up, similarly to that which is observed in typically developing children [1,4,5]. The reduction in settling time and night waking suggests improvements in night sleep consolidation and stability, reflecting ongoing neurodevelopmental maturation in this population. An increase was also observed in expressive language, as late talkers improved word and sentence production between 31 and 37 months; this result was expected even if with a slower rate compared to typically developing children [11,45].

Besides this trend of decreasing late talkers’ night sleep difficulties as perceived by parents, independently of neonatal condition, our findings showed that low-risk preterm late talkers maintained higher levels of night sleep difficulties as perceived by parents than full-term late talkers. This finding brings new evidence in support of the hypothesis that preterm infants are at a heightened risk of sleep disorders. This finding is consistent with the previous literature on the night sleep of preterm children [13,18,19] and confirms preterm children’s night sleep vulnerability. Indeed, preterm birth appears to exacerbate night sleep difficulties, even in preterm children with low neonatal immaturity and a low incidence of severe perinatal complications [13,19]. These children seem to experience more fragmented sleep and reliance on co-sleeping, potentially due to the neurodevelopmental vulnerabilities associated with prematurity [47]. Interestingly, this last evidence is in contrast with those found in the study by Lyu et al. [19], who did not find differences in the rate of co-sleeping between preterm and full-term children. This discrepancy may be attributed to cultural differences [48]. Indeed, the participants of Lyu et al.’s [19] study were from East Asian countries, where co-sleeping is a widespread parental practice, regardless of children’s age and difficulties [49,50]. Conversely, this practice is less common among parents in Western countries, where its use increases as child’s night sleep difficulties increase [50]. It is also possible that the higher rate of night sleep difficulties perceived by parents in the low-risk preterm sample than in the full-term sample depends on heightened parental distress characterizing parents of preterm children [51,52], which, in turn, may increase concerns about their infants’ sleep issues [13,53,54].

Regarding parental bedtime practices, one of the key findings was that specific parental bedtime practices, such as active physical comforting and leaving to cry, decreased in late talkers from 31 to 37 months, regardless of their neonatal condition. On the one hand, this evidence extends the existing literature on how these practices evolve and adapt in this at-risk population. The reduction in active physical comforting and leaving to cry is consistent with the developmental shift towards promoting greater autonomy as children grow up, thereby supporting more well-regulated sleep patterns [4,23]. This is consistent with practices observed in parents of typically developing peers, suggesting a common developmental and adaptive approach to fostering autonomy regardless of language mastery and neonatal condition [13]. This is a noteworthy contribution to the literature, as it highlights the importance of autonomy-promoting practices in fostering night sleep consolidation in both typically developing and at-risk populations. As regards neonatal condition, parental bedtime practices did not significantly differ between low-risk preterm and full-term late talkers except for active physical comforting, which was significantly higher in low-risk preterm late talkers than their full-term peers. This result corroborates the findings of Morrell and Cortina-Borja [23], who showed that, in children with no sleep problems, early active physical comforting practices soon decline in favour of encouraging autonomy ones, whereas in children with sleep problems, a balanced use of these two types of practices occurs later in development. According to this evidence, our results suggest that, despite the general trend towards promoting autonomy, parents of low-risk preterm late talkers tend to adapt and modulate their bedtime strategies based on their child’s needs and characteristics [13,26].

### 4.2. Associations Among Night Sleep, Parental Bedtime Practices, and Expressive Language Development in Low-Risk and Full-Term Late Talkers

Our results indicated a significant association between late talkers’ parental bedtime practices and expressive language development. Indeed, a change in parental bedtime practices, and, specifically, in encouraging autonomy bedtime practices, was positively associated with an increase in sentence production in late talkers. This result brings new evidence concerning this at-risk population that is concordant with that found in previous studies on typically developing children [26,27,42]. Parental bedtime practices of encouraging the child to settle through strategies involving talking softly, singing, or reading a story to them seem to be beneficial, not only in improving night sleep quality but also in supporting language development (e.g., [26,27,42]). These findings underscore the pivotal role of parental bedtime practices in fostering expressive language skills in late talkers during early childhood. By providing a supportive and enriched bedtime environment, parents may effectively contribute to the linguistic development of their children, thereby mitigating the adverse effects of early developmental challenges [13,26].

By contrast, no associations were found between late talkers’ night sleep and expressive language development. Several factors could explain such findings. The first concerns the developmental period considered, i.e., the second half of the third year of life. Previous studies [29,31,34] showed that the developmental trajectories of sleep are unstable during the first two years of life and characterized by a large interindividual variability that tends to decrease during the third year of life [1,4,5]. Further research is thus needed to investigate sleep trajectories in late talkers in the first two years of life and more deeply investigate the associations with later language development. Indeed, as shown by Dionne and colleagues’ study [39], sleep consolidation in the first two years of life may be a reliable marker for later language learning. The second factor to consider is related to the populations involved in the study. Previous studies [29,31,34] have examined typically developing populations or children with neurodevelopmental disorders [8], but did not focus on late talkers or consider the role of neonatal condition, i.e., preterm vs. full-term birth, both of which are the focus of our study. To obtain a complete view of the associations between night sleep and language development in late talkers born either preterm or full-term, future research comparing this at-risk population with same-aged typically developing children is needed. In addition, as night sleep is influenced by environmental and cultural factors [4,55], future research should compare late talkers and same-aged typically developing children belonging to the same culture.

### 4.3. Limitations and Future Directions

Although this study has the merit of having examined, for the first time, the developmental trajectories of night sleep and parental bedtime practices in low-risk preterm and full-term late talkers and has provided valuable insights into their relationships with language development, some limitations are worth noting.

First, this study relied on parental reports to assess night sleep and parental bedtime practices, which may be subjected to biases and inaccuracies, or indirectly lead to changes in parents’ perception of child sleep or the quantity or quality of their bedtime practices [56]. Although these kinds of measures have been largely used in previous studies involving typically and atypically developing populations [13,19], incorporating objective measures, such as actigraphy or observational assessments, could enhance the full understanding of our findings [57].

Second, the relatively small sample size prevented the analysis of night sleep patterns and parental bedtime practices based on individual profiles. As shown in some previous studies, late talkers represent a heterogeneous population characterized by distinct communicative and linguistic profiles [58,59]. Thus, different profiles might be associated with different night sleep and parental bedtime practice patterns, and these aspects might also have varying impacts on language outcomes. A future research challenge would be to examine, in concert, universal and individual differences accounting for the link between night sleep, parental bedtime practices, and language development.

Third, we only included low-risk preterm and full-term late talkers, potentially limiting the generalizability of the findings to the entire preterm population. In the future, including children born preterm with higher perinatal risk conditions and higher neonatal immaturity could provide more conclusive evidence regarding night sleep and parental bedtime practices in this population.

Fourth, our study investigated associations between night sleep, parental bedtime practices, and expressive language development in low-risk preterm and full-term late talkers in the second half of the third year of life. Future research should employ longitudinal designs, also involving earlier age points, to further elucidate the complex relationships among the above aspects. By capturing the dynamic nature of these interactions over time, researchers can gain deeper insights into the mechanisms underlying developmental trajectories in this at-risk population.

### 4.4. Clinical Implications and Conclusions

The findings of this study have significant implications for clinical practice. Understanding early night sleep trajectories and the specific challenges faced by late talkers, while also considering neonatal condition, i.e., either preterm or full-term birth, can inform the development of tailored interventions targeting this at-risk population. For example, sleep coaching programs for parents of late talkers, particularly in the case of preterm children, could focus on strategies to enhance night sleep consolidation and reduce reliance on co-sleeping. In addition, interventions targeting parental bedtime practices may offer promising avenues for promoting language development. By making parents aware of the importance of supportive parental bedtime practices, clinicians can empower caregivers to create nurturing sleep environments that also promote better linguistic outcomes [27].

In conclusion, the present study provides valuable insights into the complex relationships between night sleep, parental bedtime practices, and expressive language development in low-risk preterm and full-term late talkers. By addressing these interconnected domains holistically, clinicians can optimize outcomes across multiple domains of functioning, ultimately enhancing the overall well-being and quality of life of these at-risk children and their families.

## Figures and Tables

**Table 1:** Clinical and sociodemographic characteristics, cognitive and language scores, and age of assessment of low-risk preterm and full-term late talkers.

Participants’ Characteristics	Low-Risk Preterm Late Talkers (*n* = 19)		Full-Term Late Talkers (*n* = 19)			
	*M*/*n*	*SD*/%	*M*/*n*	SD/%	χ^2^/*t*	*p*
Gestational age (weeks), *M*, *SD*	33.05	3.20	39.63	1.12	8.47	<0.001 °
Birthweight (grams), *M*, *SD*	1833	475	3413	337	11.83	<0.001 °
Gender (male), *n*, %	18	94.7	15	78.9		0.340 ^
Firstborn, *n*, %	10	52.6	10	52.6	0.00	1.000 ^§^
Twins, *n*, %	11	57.9	0	0	15.48	<0.001 ^§^
Type of delivery (cesarean), *n*, %	15	78.9	1	5.3	21.16	<0.001 ^§^
Length of stay in hospital (days), *M*, *SD*	26.79	30.39	2.37	1.30	−3.50	0.003 °
Otitis media > 4 episodes/year, *n*, %	1	5.3	1	5.3		1.000 ^
Small for gestational age, *n*, %	3	15.8	0	0		0.230 ^
Respiratory distress syndrome, *n*, %	9	47.4	0	0		0.001 ^
Mechanical ventilation, *n*, %	2	10.5	0	0		0.486 ^
Apnea, *n*, %	1	5.3	0	0		1.000 ^
Bronchopulmonary dysplasia, *n*, %	2	10.5	0	0		0.486 ^
Retinopathy of prematurity Grade I/II, *n*, %	1	5.3	0	0		1.000 ^
Hyperbilirubinemia, *n*, %	13	68.4	0	0	19.76	<0.001 ^§^
Family history of language/learning disorders, *n*, %	2	10.5	4	21.1		0.281 ^
Childcare center attendance, *n*, %	16	84.2	17	89.5		1.000 ^
Exposure to another language, *n*, %	5	26.3	1	5.3		0.180 ^
Mother’s age (years), *M*, *SD*	39.42	5.97	37.63	5.62	−0.95	0.348 °
Father’s age (years), *M*, *SD*	40.74	7.09	39.68	6.13	−0.49	0.627 °
Mothers with high educational level (>13 years), *n*, %	12	63.2	13	68.4	0.117	0.732 ^§^
Fathers with high educational level (>13 years), *n*, %	8	42.1	12	63.2	1.689	0.194 ^§^
Mother’s nationality (Italian), *n*, %	15	78.9	18	94.7		0.340 ^
Father’s nationality (Italian), *n*, %	14	73.7	19	100		0.046 ^
BSID-III cognitive composite score, *M*, *SD*	87.37	7.52	86.76	7.49	−0.241	0.811 °
BSID-III language composite score, *M*, *SD*	81.18	8.18	81.07	11.97	−0.031	0.976 °
Age of assessment at 31 months *, months, *M*, *SD*	31.48	1.41	31.34	1.41	−0.293	0.771 °
Age of assessment at 37 months *, months, *M*, *SD*	37.46	1.92	37.71	1.21	0.489	0.628 °

Note: See [13,44] for a detailed description of the medical complications reported in Table 1. Significant results are reported for the *t*-test (°), Chi-square test (§), and Fisher’s exact test (^) when at least one expected value was <0.05. * Age was corrected for low-risk preterm late talkers. Missing data: BSID-III cognitive composite score: *n* = 2 full-term children; BSID-III language composite score: *n* = 2 low-risk preterm children, *n* = 4 full-term children.

**Table 2:** Descriptive statistics and repeated measure analyses of variance of low-risk preterm and full-term late talkers’ ISQ (Infant Sleep Questionnaire) scores at 31 and 37 months.

		Low-Risk Preterm Late Talkers (*n* = 19)								Full-Term Late Talkers (*n* = 19)						
	*M*	*SD*	*Range*	*M*	*SD*	*Range*	*M*	*SD*	*M*	*SD*	*Range*	*M*	*SD*	*Range*	*M*	*SD*
	31 months			37 months			Δ	Δ	31 months			37 months			Δ	Δ
ISQ																
Total score	13.42	5.89	1–26	8.53	5.32	0–21	−4.89	7.34	6.61	7.84	1–32	5.44	4.97	0–16	−1.17	4.57
Settling	3.26	3.11	0–10	1.84	1.86	0–6	−1.42	2.24	2.84	3.15	0–11	2.58	2.48	0–7	−0.26	1.85
Night waking	5.89	3.67	0–10	3.95	3.73	0–10	−1.95	4.61	2.56	3.81	0–14	2.11	3.01	0–9	−0.44	1.82
Co-sleeping	4.26	3.41	0–7	2.74	3.28	0–7	−1.53	3.89	1.67	2.54	0–7	1.00	1.88	0–7	−0.67	3.11
			*F*						*p*			*η* *_p_* ^2^				
Total score																
Age			8.98						0.005			0.204				
Group			8.18						0.007			0.189				
Age x group			3.40						0.074			0.088				
Settling																
Age			6.37						0.016			0.150				
Group			0.04						0.847			0.001				
Age x group			3.01						0.091			0.077				
Night waking																
Age			4.21						0.048			0.107				
Group			6.44						0.016			0.155				
Age x group			1.66						0.206			0.045				
Co-sleeping																
Age			3.56						0.067			0.092				
Group			8.56						0.006			0.196				
Age x group			0.55						0.464			0.015				

Note: Missing data ISQ: total score at 31 months, *n* = 1 full-term child; night waking at 31 months, *n* = 1 full-term child; co-sleeping at 31 months, *n* = 1 full-term child.

**Table 3:** Descriptive statistics and repeated measure analyses of variance of low-risk preterm and full-term late talkers’ PIBBS (Parental Interactive Bedtime Behavior Scale) scores at 31 and 37 months.

		Low-Risk Preterm Late Talkers (*n* = 19)								Full-Term Late Talkers (*n* = 19)						
	*M*	*SD*	*Range*	*M*	*SD*	*Range*	*M*	*SD*	*M*	*SD*	*Range*	*M*	*SD*	*Range*	*M*	*SD*
	31 months			37 months			Δ	Δ	31 months			37 months			Δ	Δ
PIBBS																
Total score	24.11	10.06	8–39	22.84	8.43	10–41	−1.26	8.72	22.00	10.51	4–48	19.32	7.82	2–33	−2.68	7.47
Active physical comforting	10.74	5.19	4–19	9.00	4.01	1–19	−1.74	4.92	7.58	6.74	0–28	6.00	4.16	0–18	−1.58	4.38
Encouraging autonomy	12.79	7.74	0–23	13.79	6.09	2–22	1	5.93	14.11	5.01	4–24	13.32	5.63	0–21	−0.79	4.37
Leaving to cry	0.58	1.17	0–3	0.05	0.23	0–1	−0.53	1.12	0.32	0.82	0–3	0.00	0.00	0–0	−0.32	0.82
			*F*						*p*			*η* *_p_* ^2^				
Total score																
Age			1.08						0.305			0.029				
Group			2.25						0.143			0.059				
Age x group			0.29						0.593			0.008				
Active physical comforting																
Age			4.82						0.035			0.118				
Group			4.28						0.046			0.106				
Age x group			0.01						0.917			0.000				
Encouraging autonomy																
Age			0.02						0.902			0.000				
Group			0.05						0.819			0.001				
Age x group			1.12						0.297			0.030				
Leaving to cry																
Age			6.96						0.012			0.162				
Group			0.84						0.365			0.023				
Age x group			0.44						0.514			0.012				

**Table 4:** Descriptive statistics and repeated-measure analyses of variance of low-risk preterm and full-term late talkers’ word and sentence production MB-CDIs (MacArthur-Bates Communicative Development Inventories) scores at 31 and 37 months.

		Low-Risk Preterm Late Talkers (*n* = 19)								Full-Term Late Talkers (*n* = 19)						
	*M*	*SD*	*Range*	*M*	*SD*	*Range*	*M*	*SD*	*M*	*SD*	*Range*	*M*	*SD*	*Range*	*M*	*SD*
	31 months			37 months			Δ	Δ	31 months			37 months			Δ	Δ
MB-CDI																
Word production	146.42	115.10	15–505	289.42	212.92	19–678	143.00	144.79	96.00	85.10	7–269	279.53	215.41	12–610	183.53	166.91
Sentence production	13.53	15.61	0–37	25.26	15.14	0–37	11.79	13.24	8.16	11.16	0–36	23.16	14.91	0–37	15.00	13.17
			*F*						*p*			*η* *_p_* ^2^				
Word production																
Age			41.49						<0.001			0.535				
Group			0.39						0.534			0.011				
Age x group			0.64						0.429			0.017				
Sentence production																
Age			38.96						<0.001			0.520				
Group			0.82						0.371			0.022				
Age x group			0.58						0.451			0.016				

**Table 5:** Pearson’s correlation coefficients between 31- and 37-month change (deltas, Δ) in low-risk preterm and full-term late talkers’ ISQ (Infant Sleep Questionnaire) scores, PIBBS (Parental Interactive Bedtime Behavior Scale) scores, and word and sentence production MB-CDIs (MacArthur-Bates Communication Development Inventories) scores.

	Δ Word Production MB-CDI Score		Δ Sentence Production MB-CDI Score	
	*r*	*p*	*r*	*p*
Δ ISQ total score	0.100	0.955	−0.027	0.873
Δ Settling	0.024	0.888	0.163	0.327
Δ Night waking	−0.053	0.755	−0.098	0.563
Δ Co-sleeping	0.079	0.641	−0.039	0.820
Δ PIBBS total score	−0.017	0.920	0.346	0.033
Δ Active physical comforting	−0.147	0.377	0.201	0.226
Δ Encouraging autonomy	0.063	0.705	0.416	0.009
Δ Leaving to cry	0.216	0.192	−0.318	0.052

## Data Availability

The dataset presented in this article is not readily available because it includes sensitive information about minors with developmental vulnerabilities. Requests to access the dataset should be directed to the corresponding author.

## Supplementary Materials

The following supporting information can be downloaded at: https://www.mdpi.com/article/10.3390/children11111393/s1.
