# Supplementary material for: Night Sleep, Parental Bedtime Practices and Language Development in Low-Risk Preterm and Full-Term Late Talkers: A Longitudinal Study in the Third Year of Life
Source: Children (Basel). 2024 Nov 16;11(11):1393. doi: 10.3390/children11111393 (PMC11592692; doi:10.3390/children11111393)
Supplement: Supplementary file 1 [file children-11-01393-s001.zip › Supplementary_Materials_Children_Zuccarini_et_al_2024.pdf]

Supplementary Materials

Article

Night Sleep, Parental Bedtime Practices and Language Development in Low-Risk Preterm and Full-Term Late Talkers: A Longitudinal Study in the Third Year of Life

Mariagrazia Zuccarini [1], Martina Riva [2,3], Arianna Aceti [4,5], Luigi Corvaglia [4,5], Anat Scher [6], Annalisa Guarini [2] and Alessandra Sansavini [2,*]

[1] Department of Education Studies "Giovanni Maria Bertin", University of Bologna, Via Filippo Re 6, 40126 Bologna, Italy; mariagrazia.zuccarini@unibo.it
[2] Department of Psychology "Renzo Canestrari", University of Bologna, Viale Berti Pichat 5, 40127 Bologna, Italy; martina.riva4@studio.unibo.it (M.R.); annalisa.guarini@unibo.it (A.G.)
[3] Department of Psychology, University of Milano-Bicocca, Piazza dell'Ateneo Nuovo 1, 20126 Milano, Italy
[4] Neonatal Intensive Care Unit, IRCCS Azienda Ospedaliero-Universitaria di Bologna, Via Massarenti 9, 40138 Bologna, Italy; arianna.aceti2@unibo.it (A.A.); luigi.corvaglia@unibo.it (L.C.)
[5] Department of Medical and Surgical Sciences, University of Bologna, Via Massarenti 9, 40138 Bologna, Italy
[6] Department of Counseling and Human Development, University of Haifa, Abba Khoushy Ave 199, Haifa 3498838, Israel; anats@edu.haifa.ac.il
* Correspondence: alessandra.sansavini@unibo.it

SUPPLEMENTARY MATERIALS

SA. Questionario sul Sonno nella Prima Infanzia

Italian version of the Infant Sleep Questionnaire—ISQ (for the English version, see Morrell, J.M.B. The Infant Sleep Questionnaire: A new tool to assess infant sleep problems for clinical and research purposes. *Child Psychol. Psyc. Rev.* **1999**, 4, 26. https://doi.org/10.1017/S1360641798001816; for details of the Italian version, see Sansavini, A.; Riva, M.; Zuccarini, M.; Aceti, A.; Corvaglia, L.; Scher, A.; Guarini, A. Night sleep and parental bedtime practices in low-risk preterm and full-term late talkers. *Children* **2022**, 9, 1813. https://doi.org/10.3390/children9121813.

Gentile Genitore, nel questionario trova alcune domande sulle abitudini di sonno del/la suo/a bambino/a.
Per favore risponda alle domande sulla base di quanto ha osservato nelle ultime due settimane.

Addormentamento

1.	Quanto tempo di solito impiega mediamente per addormentare il/la suo/a bambino/a? (segni con una X una sola risposta)	
	[ ]	Meno di 10 minuti
	[ ]	da 10 a 20 minuti
	[ ]	da 20 a 30 minuti
	[ ]	da 30 a 40 minuti
	[ ]	da 40 a 50 minuti
	[ ]	da 50 a 60 minuti
	[ ]	1 ora o più a lungo
2.	Quante volte alla settimana mediamente ha difficoltà ad addormentare il/la suo/a bambino/a? (segni con una X una sola risposta)	
	[ ]	Difficoltà meno di una volta a settimana
	[ ]	Difficoltà 1 notte a settimana
	[ ]	Difficoltà 2 notti a settimana
	[ ]	Difficoltà 3 notti a settimana
	[ ]	Difficoltà 4 notti a settimana
	[ ]	Difficoltà 5 notti a settimana
	[ ]	Difficoltà 6 notti a settimana
	[ ]	Difficoltà ogni notte della settimana
*3.	Da quanto tempo si manifestano le difficoltà di addormentamento del/la suo/a bambino/a? ……… mesi<br>[ ] non applicabile	

Risvegli notturni (tra mezzanotte e le 6 del mattino)

4.	Quante notti a settimana si sveglia mediamente il/la suo/a bambino/a? (segni con una X una sola risposta)	
	[ ]	Mai o meno di una volta a settimana
	[ ]	1 notte a settimana
	[ ]	2 notti a settimana
	[ ]	3 notti a settimana
	[ ]	4 notti a settimana
	[ ]	5 notti a settimana
	[ ]	6 notti a settimana
	[ ]	Ogni notte della settimana
5.	Quante volte si sveglia mediamente ogni notte il/la suo/a bambino/a e ha bisogno di essere riaddormentato/a? (segni con una X una sola risposta)	
	[ ]	Non si sveglia
	[ ]	1 volta per notte
	[ ]	2 volte per notte

	[ ]	3 volte per notte
	[ ]	4 volte per notte
	[ ]	5 o più volte per notte
6.	Se il/la suo/a bambino/a si sveglia, quanto tempo impiega mediamente a riaddormentarsi? (segni con una X una sola risposta)	
	[ ]	Meno di 10 minuti
	[ ]	da 10 a 20 minuti
	[ ]	da 20 a 30 minuti
	[ ]	da 30 a 40 minuti
	[ ]	da 40 a 50 minuti
	[ ]	da 50 a 60 minuti
	[ ]	1 ora o più
*7.	Da quanto tempo si manifesta il problema dei risvegli notturni?<br>……… mesi [ ] non applicabile	

Dormire nel letto dei genitori

8.	Quanto spesso porta il/la suo/a bambino/a nel suo letto perché è agitato/a e non riesce a dormire? (segni con una X una sola risposta)	
	[ ]	Mai, o meno di una volta alla settimana
	[ ]	1 notte a settimana
	[ ]	2 notti a settimana
	[ ]	3 notti a settimana
	[ ]	4 notti a settimana
	[ ]	5 notti a settimana
	[ ]	6 notti a settimana
	[ ]	Ogni notte della settimana
*9.	Da quanto tempo si manifesta questo problema?<br>……… mesi [ ] non applicabile	

Il suo punto di vista

*10.	Pensa che il/la suo/a bambino/a abbia difficoltà di sonno?<br>(segni con una X una sola risposta)	
	[ ]	No
	[ ]	Sì, lievi
	[ ]	Sì, moderate
	[ ]	Sì, severe

* *Note*. Items 3, 7, 9, and 10 do not contribute to the calculation of the ISQ scores and were not used in the current study.

SB. Scala sui Comportamenti Interattivi dei Genitori per l'Addormentamento

Italian version of the Parental Interactive Bedtime Behavior Scale—PIBBS (for the English version, see Morrell, J.; Cortina-Borja, M. The Developmental Change in Strategies Parents Employ to Settle Young Children to Sleep, and Their Relationship to Infant Sleeping Problems, as Assessed by a New Questionnaire: The Parental Interactive Bedtime Behaviour Scale. *Infant Child Dev*. **2002**, 11, 17–41. https://doi.org/10.1002/icd.251; for details of the Italian version, see Sansavini, A.; Riva, M.; Zuccarini, M.; Aceti, A.; Corvaglia, L.; Scher, A.; Guarini, A. Night sleep and parental bedtime practices in low-risk preterm and full-term late talkers. *Children* **2022**, 9, 1813. https://doi.org/10.3390/children9121813..

Gentile Genitore,
Quali metodi usa per aiutare il/la suo/a bambino/a ad addormentarsi?
Quanto spesso usa ciascuno di questi metodi?
Per favore, risponda alle domande sulla base dei comportamenti che ha messo in atto nelle ultime due settimane per addormentare il/la suo/a bambino/a; segni con una X le risposte appropriate, una sola risposta per riga.

Mai 0 Raramente 1 Qualche volta 2 Spesso 3 Molto spesso 4	0	1	2	3	4
1. Accarezzare il/la bambino/a o dargli dei colpetti					
2. Tenere in braccio o dondolare il/la bambino/a in braccio					
3. Portare il/la bambino/a in braccio in giro per la casa					
4. Portare il/la bambino/a in giro in carrozzina o in passeggino					
5. Far fare al/la bambino/a un giro in macchina					
6. Far ascoltare al/la bambino/a musica o giochi musicali					
7. Parlare dolcemente al/la bambino/a					
8. Cantare al/la bambino/a una ninna nanna					
9. Leggere una storia al/la bambino/a					
10. Giocare con il/la bambino/a					
11. Dare al/la bambino/a il suo oggetto o pezzo di stoffa preferito					
12. Dare al/la bambino/a da mangiare o da bere					
13. Lasciare piangere il/la bambino/a					
14. Stare vicino al letto/culla del/la bambino/a senza prenderlo/a in braccio					
15. Addormentare il/la bambino/a sul divano con il genitore					
16. Stendersi con il/la bambino/a vicino al suo letto/culla					
17. Addormentare il/la bambino/a nel letto dei genitori					
